# A raw deal: the intersection of the immunocompromised cat, influenza, and a lapse of infection prevention and control measures

**DOI:** 10.1128/asmcr.00180-25

**Published:** 2025-12-03

**Authors:** Kelli J. Maddock

**Affiliations:** 1 Veterinary Diagnostic Laboratory, North Dakota State University 3323, Fargo, North Dakota, USA; Rush University Medical Center, Chicago, Illinois, USA

## Abstract

The case report by Chen et al. (ASM Case Rep 1:e00134-25, 2025, https://doi.org/10.1128/asmcr.00134-25) importantly describes nosocomial transmission of highly pathogenic avian influenza (HPAI) in an immunocompromised resident clinic cat. This case highlights several significant public health threats, starting with HPAI spillover from birds to cats, foxes, and cattle; probable HPAI infection due to contaminated commercially prepared raw pet foods; nosocomial infection of an immunocompromised animal; and lapses in infection prevention and control (IPC) measures. This case underscores the need for formal IPC guidelines or a risk assessment-based protocol for resident clinic or personal pets in veterinary settings. As the availability of and interest in using commercial raw pet foods increase, there is a need for education surrounding infectious disease risk, safe handling, and handwashing procedures for use of these products, as well as the potential risk for fecal shedding of important enteric pathogens. Further emphasizing the importance of IPC, nosocomial outbreaks caused by *Salmonella* and antimicrobial-resistant bacteria have been identified in large animal and companion animal practices, with a recent case describing probable zoonotic and nosocomial spread of carbapenem-resistant organisms. To mitigate these risks, veterinary practices (clinics or hospitals) require resources and education for IPC to minimize and investigate nosocomial transmission of infectious diseases. Finally, the availability of high-quality point-of-care test options for veterinary infectious diseases would support human, animal, and population health by providing rapid and actionable results for disease response.

## COMMENTARY

The case report by Chen et al. (1) describes a nosocomial infection of highly pathogenic avian influenza (HPAI) H5N1 in an immunocompromised domestic cat. The cat was a resident of the clinic and had contact with clinic spaces and personnel who had previous contact with another cat experiencing respiratory illness after consumption of recalled (2), potentially HPAI-contaminated raw cat food. This case represents an interesting intersection of an immunocompromised resident of a veterinary clinic and several public health phenomena of our time, namely, the spillover of HPAI from birds to cats, foxes, and cattle; the raw pet food diet trend; and an increasing need for infection prevention and control (IPC) measures in veterinary practices (clinics or hospitals). Beyond these factors, human, animal, and population health, and potentially antimicrobial stewardship could be supported by increased availability of high-quality infectious disease point-of-care testing (POCT) in veterinary practices.

It is not uncommon for veterinary practices to adopt resident clinic pets with difficult to manage conditions that may be too challenging or expensive for the average pet owner to navigate. While surrounding these animals with veterinary professionals and providing care is altruistic, paradoxically, they are also residing in an environment where they may be at a high risk of contracting a debilitating or lethal illness from other patients. While no formal IPC guidance or policy currently exists for personal or clinic-residing pets, there is a significant biosecurity risk and the potential for infectious disease spread for these animals, particularly if they are also immunocompromised. In 2019, the American Society for Microbiology drafted formal guidance for service animals in teaching laboratory settings (3), with emphasis placed on protecting both the animal and people in the space from physical harm (trips and uncontrolled animal), biohazards, and biosecurity risks. Veterinary practices should evaluate potential risks to these resident clinic pets and whether procedural control or physical segregation from common areas that may be contaminated by other patients is sufficient to protect these vulnerable animals.

In recent years, a trend toward providing raw pet food for perceived nutrition benefits (4–6) has become a more mainstream dietary option for pets, with multiple commercial vendors offering raw products marketed to pet owners. Despite numerous infectious disease events (7) and decades of evidence to the contrary (8), raw milk is also experiencing a new era of public perception as a nutritious, safe, and natural alternative to pasteurized dairy products for people and pets alike. Commercial raw pet foods have also been the subject of product recalls (2, 9, 10) due to potential HPAI contamination, and both raw pet foods and raw milk have been linked to HPAI infection in companion animals, including the probable source of HPAI in this case (1). While the risk of cat to human transmission of HPAI is currently low, raw diets introduce additional risks to pets and their owners. These products are often contaminated with ever-present gastrointestinal nemeses, *Salmonella, Listeria monocytogenes, Campylobacter,* and diarrheagenic *Escherichia coli* (4, 5, 11–13), with a recent outbreak involving raw pet food and a multidrug-resistant strain of *Salmonella* I 4, [5], 12:i:- (14). Pet owners may not realize that these raw commercial products require strict handling, just like human-grade meat products that are typically cooked. They also may be unaware of the risks associated with these raw products. Beyond product handling risk, there is an increased risk of fecal *Salmonella* shedding from pets who have consumed contaminated products (15, 16), increasing the risk of illness for pet owners or environmental contamination. Veterinary professionals, retailers, and commercial raw pet food companies share the responsibility to communicate these potential risks and provide safe handling resources to pet owners.

Historically, data regarding hospital-acquired infections (HAIs) and IPC have been limited for veterinary medicine (17). Interactions with veterinary patients may seem less clinical than interactions with human patients, and hand hygiene, personal protective equipment use, and equipment or environmental decontamination may not be prioritized in some clinical settings until there is an adverse event requiring it to be. While documentation of HAI is scarce for most pathogens, the primary suspects in human HAIs are also of concern for veterinary settings (17). *Salmonella* outbreaks (17–19) are well documented in large animal hospitals, and outbreaks caused by carbapenemase-producing organisms (CPOs) in companion animal practices were recently described in the United States (20–23). While it is possible that these CPO outbreaks are new phenomena, it is also possible that detection and reporting of these strains have been delayed or limited due to lack of veterinary laboratory resources, knowledge, or procedures for public health reporting of these isolates from animals (24). Concerningly, a recent study (20) epidemiologically linked three human cases of infection with a CPO to a veterinary hospital where pets residing in these three separate households had received care, further highlighting the zoonotic and nosocomial risk of these outbreaks. Beyond risk to patients and their families, HAIs are expensive for veterinary practices and their patients with cost estimates ranging from $25,000 for mitigation of a CPO outbreak (21) to $4.12 million in lost revenue and mitigation expenses (19) for a multidrug-resistant *Salmonella* outbreak. Importantly, lapses of IPC measures were identified in each *Salmonella* and CPO outbreak report.

Finally, veterinary medicine would benefit from the availability of high-quality, rapid, infectious disease POCT (25), such as PCR panels for common viral, bacterial, and parasitic causes of illness like those employed in human medical settings. Rapid respiratory POCT could help rule in or out influenza and common viral or bacterial causes of respiratory illness, supporting rapid treatment and response to infectious disease. In the case of the initially presenting feline, a rapid influenza result may have prompted decontamination procedures and IPC measures, protecting animal population health. Again, while the risk of cat to human transmission of HPAI currently remains low, rapid detection of critical emerging infectious diseases from humans or animals is important in the post-pandemic watchful waiting period. Beyond infectious disease detection and transmission, rapid POCT could also enhance antimicrobial stewardship by supporting veterinarians in conversations with pet owners where antimicrobial treatments are requested by an owner for what is often a viral infection.

Particularly in a time of wavering trust in public health (26), it is important for veterinarians and other health professionals to continue the low-cost preventative strategy of sharing the science and educating animal owners and the general public regarding the risks of raw diets for pets. Owners must be aware of the potential risks of using raw products for themselves and their animals, and veterinarians must feel empowered to discuss these risks as well as the need for strict handling and handwashing measures undertaken by the owners. Veterinary practices must have resources and support for IPC measures, including training for appropriate hand hygiene and personal protective equipment use, decontamination practices, and outbreak investigations. In the absence of formal IPC guidance or policies, veterinary practices should conduct risk assessments for personal and resident clinic pets. Resources should be allocated for the development of high-quality POCT to ensure timely detection of infectious disease agents. Together, these measures will support IPC and safeguard human and animal health.
